# Dr. Ogilvie's Course of Lectures on Medical Psychology

**Published:** 1850-10-01

**Authors:** 


### DR. OGILVIE'S COURSE OF LECTURES ON MEDICAL PSYCHOLOGY.

The study of medical psychology appears to be attracting the attention it deserves as a branch of professional education. Of this we have a pleasing proof before us, in the list of the medical classes taught in the University and Marischal College, Aberdeen, which includes a course of twenty-five lectures on mental disease, by Dr. J. F. Ogilvie, the resident physician of the Lunatic Asylum of that place.

So far as we are aware, this is the first occasion on which any of our British universities or schools of medicine have provided for the instruction of their students in this special and important department of practice, and we sincerely trust that the success which it appears to have attained will induce others to follow up the spirited example thus set before them. The plan pursued by Dr. Ogilvie, as we gather from the outline of his course, with which we have been favoured, seems to us to be admirably adapted for the end in view; and were our examining boards to enforce the attendance of our students on such a course of lectures, we should augur most favourably for the rapid and steady advance of this interesting, but hitherto much neglected branch of medical science.

